# Mechanistic Insights into CO_2_ Adsorption of Li_4_SiO_4_ at High Temperature

**DOI:** 10.3390/ma18020319

**Published:** 2025-01-12

**Authors:** Nan Ma, Silin Wei, Jinglin You, Fu Zhang, Zhaohui Wu

**Affiliations:** 1 Hunan Key Laboratory of Applied Environmental Photocatalysis, School of Materials and Environmental Engineering, Changsha University, Changsha 410022, China; w1625272361@163.com (S.W.); hubeiwzh1624@126.com (Z.W.); 2 State Key Laboratory of Advanced Special Steel, Shanghai Key Laboratory of Advanced Ferrometallurgy, School of Materials Science and Engineering, Shanghai University, Shanghai 200444, China; jlyou@163.com; 3 State Key Laboratory of Applied Organic Chemistry, Key Laboratory of Nonferrous Metal Chemistry and Resources Utilization of Gansu Province, College of Chemistry and Chemical Engineering, Lanzhou University, Lanzhou 730000, China; fuzhang201026@163.com

**Keywords:** CO_2_ adsorbent, in situ X-ray diffraction, in situ Raman spectroscopy, adsorption mechanism

## Abstract

The development of materials with high adsorption capacity for capturing CO_2_ from industrial exhaust gases has proceeded rapidly in recent years. Li_4_SiO_4_ has attracted attention due to its low cost, high capture capacity, and good cycling stability for direct high-temperature CO_2_ capture. Thus far, the CO_2_ adsorption mechanism of Li_4_SiO_4_ is poorly understood, and detailed phase transformations during the CO_2_ adsorption process are missing. Here, aided by in situ X-ray diffraction and in situ Raman spectroscopy, we find that Li_4_SiO_4_ reacts with CO_2_ to form Li_2_SiO_3_ and Li_2_CO_3_ in CO_2_ atmosphere at 973 K, with no detectable involvement of crystalline Li_2_O during the adsorption process. Moreover, we observe a formation of stepped structures in the Li_4_SiO_4_ surface after CO_2_ adsorption by scanning electron microscopy. To illustrate the formation of stepped structures, we propose a modified double-shell mechanism, suggesting a possible two-dimensional nucleation and growth of Li_2_CO_3_. This work provides a deeper understanding of the CO_2_ adsorption mechanism and paves a way for further optimization of Li_4_SiO_4_-based adsorbents.

## 1. Introduction

Carbon dioxide (CO_2_), a primary greenhouse gas emitted from industrial activities and daily life, significantly impacts global climate and environmental systems [1]. Employing CO_2_ adsorbents can effectively mitigate CO_2_ emissions in industrial processes, and the captured CO_2_ serve as a valuable carbon resource with diverse applications across various sectors of the national economy, including uses as chemical raw materials, refrigerants, oilfield stimulation agents, inert solvents, and pressure sources [2,3,4].

Extensive research has been reported on using activated carbon [5], organic amines [6], calcium-based materials [7], and lithium-based materials [8] as potential candidates for CO_2_ adsorbents. Among them, lithium orthosilicate (Li_4_SiO_4_) stands out due to its low cost, high capture capacity, and good cycling stability for direct high-temperature CO_2_ capture, making it an ideal adsorbent for separating CO_2_ from flue gases [9,10,11,12,13,14]. Ever since the proposal of Li_4_SiO_4_ as a high-temperature CO_2_ adsorbent by Kato et al. [15] in 2002, numerous studies have emerged mainly focusing on improving CO_2_ adsorption performance though condition control [16,17,18], impurity doping [19,20], and structural improvements [21,22,23].

To further enhance CO_2_ adsorption performance, understanding the adsorption mechanism is crucial. Kato et al. [15,22] suggested that the adsorption process involves a reversible reaction between Li_2_O in the Li_4_SiO_4_ crystal structure and CO_2_. They pointed out that the diffusion of Li_2_O to the Li_4_SiO_4_ surface is considered as the rate-limiting step at a higher CO_2_ concentration, while the surface reaction becomes the rate-limiting step for lower CO_2_ concentrations [24]. Zhang et al. [17,18] proposed a double-shell mechanism to explain the reaction of Li_4_SiO_4_ with CO_2._ They declared that the products, Li_2_SiO_3_ and Li_2_CO_3_, cover the unreacted Li_4_SiO_4_, separating into two product layers, with the Li_2_SiO_3_ being the inner layer and the Li_2_CO_3_ the outer layer [17,18]. Subsequently, the unreacted Li_4_SiO_4_ generates O^2−^ and Li^+^ ions, which diffuse outward through the Li_2_SiO_3_ shell and react with CO_2_ that has diffused inward through the Li_2_CO_3_ shell [17,18]. Most recently, though creative inert-marker experiments, Deng et al. [25] reported that the dominant diffusion mode in the product layers only involves the outward diffusion of O^2−^ and Li^+^ ions rather than the diffusion of Li_2_O or CO_2_, and the O^2−^ ion first diffuses outward to the surface and reacts with CO_2_ to form CO_3_^2−^, while Li^+^ ions diffuse outward and react with CO_3_^2−^ to form Li_2_CO_3_. However, to further confirm the CO_2_ adsorption mechanism of Li_4_SiO_4_, the in situ observations [26,27,28] of phase transformations during the CO_2_ adsorption process still remain to be examined.

In this work, we carried out a comprehensive investigation of the CO_2_ capture performances of Li_4_SiO_4_ adsorbent and the associated phase transformations during the CO_2_ adsorption process. This was achieved through a combination of thermogravimetric (TG) analysis, in situ X-ray diffraction (XRD), in situ Raman spectroscopy analysis, and density functional theory (DFT) calculations. Additionally, scanning electron microscopy (SEM) was utilized to characterize the surface morphology of the Li_4_SiO_4_ adsorbent both before and after CO_2_ adsorption. Finally, we proposed a modified double-shell model to elucidate the underlying mechanism of the CO_2_ adsorption process.

## 2. Materials and Methods

### 2.1. Material Synthesis

Analytical reagent grade Li_2_CO_3_ and SiO_2_, both sourced from Sinopharm Chemical Reagent Co., Ltd., Shanghai (China), were used in the present work. All the chemicals were dried at 473 K for 2 h to remove the surface moisture. Stoichiometric amounts of Li_2_CO_3_ and SiO_2_ were initially mixed and ground in an agate mortar with an appropriate amount of ethanol for 1 h, then transferred into a corundum crucible. The mixture was synthesized at a temperature of 1123 K for a period of 6 h in an air atmosphere. The resultant products were subsequently ground for further analyses.

### 2.2. Material Characterization

The in situ X-ray powder diffraction (XRD) measurements were conducted using the Bruker D8 Advance X-ray diffractometer (Bruker AXS, Karlsruhe, Germany) (40 KV, 40 mA) with Cu/Kα1 radiation (*λ* = 0.15406 nm). In the heating process, the patterns were recorded in N_2_ atmosphere in the 2θ range of 10° to 90° with a step size of 0.02° and a counting time of 0.1 s per step.

The in situ Raman spectroscopy measurements were carried by using a Horiba Jobin Y’von LabRAM HR Evolution Raman spectrometer (Horiba Jobin Y’von, Paris, France). The microscopic heating furnace was a Linkam TS1500 (Linkam, Tadworth, UK) with a temperature deviation within ±1 K. The laser wavelength is 532 nm (Coherent, Santa Clara, CA, USA), and the integration time is 10 s 10 time.

The in situ XRD and in situ Raman spectroscopy have the same heating program. The heating rate is 10 K/min, with a N_2_ flow rate of 45 cm^3^/min. When the temperature rises to 973 K, the atmosphere changes from N_2_ to CO_2_, holding for 150 min. During the heat preservation period, the spectrum was measured every ten minutes.

The thermogravimetric (TG) analyses were performed by the Netzsch STA449F5 simultaneous thermal analyzer (Netzsch, Stuttgart, Germany). During the initial heating process, both the protective and purge gases were N_2_, with a gas flow rate of 20 cm^3^/min and a heating rate of 10 K/min. When the temperature reached 973 K, the purge gas was switched from N_2_ to CO_2_, and the flow rate for both the protective and purge gases was increased to 45 cm^3^/min. The system was then maintained at this temperature for 150 min.

A Zeiss Sigma 300 scanning electron microscope (SEM) (Carl Zeiss AG, Oberkochen, Germany) was used to determine the morphology of Li_4_SiO_4_ particles before and after CO_2_ adsorption. All samples were sputter-coated with Au before the measurements due to the poor electron conductivity of Li_4_SiO_4_.

### 2.3. Computational Details

Density functional theory (DFT) calculations were performed using the Cambridge Serial Total Energy Package (CASTEP) code (Materials Studio 8.0) [29,30]. The Raman wavenumber and intensity of various vibration modes of Li_4_SiO_4_, Li_2_SiO_3_, and Li_2_CO_3_ crystals were used by the functional set LDA (CA−PZ) version [31,32] with optimized norm-conserving pseudopotentials [33]. A plane wave energy cut-off of 600 eV was used for all computations. The *k*-point meshes were set as 3 × 3 × 3 for all structures. The convergence criterion for the electronic self-consistent field was set to 2 × 10^−6^ eV, and the maximum atomic force was determined to be 0.02 eV/Å. All the initial crystal structures were obtained from the Inorganic Crystal Structure Database (ICSD) [34].

## 3. Results and Discussion

### 3.1. Characterization of the Pristine Li_4_SiO_4_

**Figure 1:**
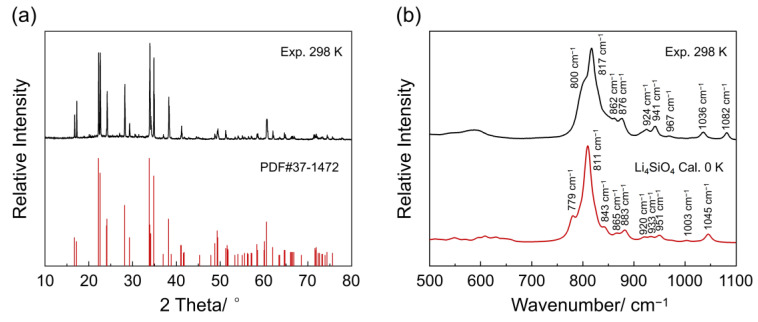
(**a**) XRD pattern of pristine Li_4_SiO_4_. (**b**) Calculated and measured Raman spectra of pristine Li_4_SiO_4_.

Figure 1a shows the room-temperature XRD pattern of the pristine Li_4_SiO_4_ sample. The diffraction peaks of pristine Li_4_SiO_4_ corresponded well with the main reflections at 2θ = 22.2°, 22.6°, 33.8°, and 34.8° of the standard monoclinic phase of Li_4_SiO_4_, demonstrating the successful preparation of Li_4_SiO_4_. In addition, it is noteworthy that no XRD diffraction peaks for Li_2_O are observed.

Li_4_SiO_4_ crystallizes in the monoclinic *P*2_1_/*m* space group. The unit cell of Li_4_SiO_4_ (ICSD No. 35169) contains fourteen formula units (Li_8_Si_2_O_8_) with lattice *a* = 5.147 Å, *b* = 6.094 Å, *c* = 5.293 Å, *α* = 90°, *β* = 90.33°, *γ* = 90°, and *V* = 166.017 Å^3^ [35]. Figure S1 (see the supplementary materials) shows the unit cell of Li_4_SiO_4_ which contains two SiO_4_ tetrahedra which are isolated from one another. Based on the crystal structure of Li_4_SiO_4_, we obtained the theoretical Raman spectrum of Li_4_SiO_4_ by DFT calculations. As shown in Figure 1b, there are five main peaks observed at 779, 811, 883, 951, and 1045 cm^−1^ in the theoretical spectrum. Notably, the bands at 779, 811, 883, and 1045 cm^−1^ correspond to the symmetrical stretching vibrational modes of the Si–O bonds of isolated SiO_4_ tetrahedra. In addition, the band located at 951 cm^−1^ belongs to the rocking vibration modes of the Si–O bonds. The major calculated vibrational modes of the Li_4_SiO_4_ crystal are listed in Table 1. In addition, the peak shapes of the measured Raman spectra of pristine Li_4_SiO_4_ are consistent with previous results [14]. Five peaks at 800, 817, 876, 967, and 1082 cm^−1^ are assigned to Li_4_SiO_4_, according to the theoretical Raman spectrum. In addition, according to previous reports, if Li_2_O exists, its peak might appear around 534 cm^−1^ [36]. However, in the experimental Raman spectrum of pristine Li_4_SiO_4_, no peaks corresponding to Li_2_O are observed. Notably, the overall peaks of the theoretical Raman spectrum are slightly lower than those of the experimental spectrum, which could be attributed to the DFT calculations being conducted at 0 K, without taking into account the influence of temperature.

### 3.2. CO_2_ Capture Performance

**Figure 2:**
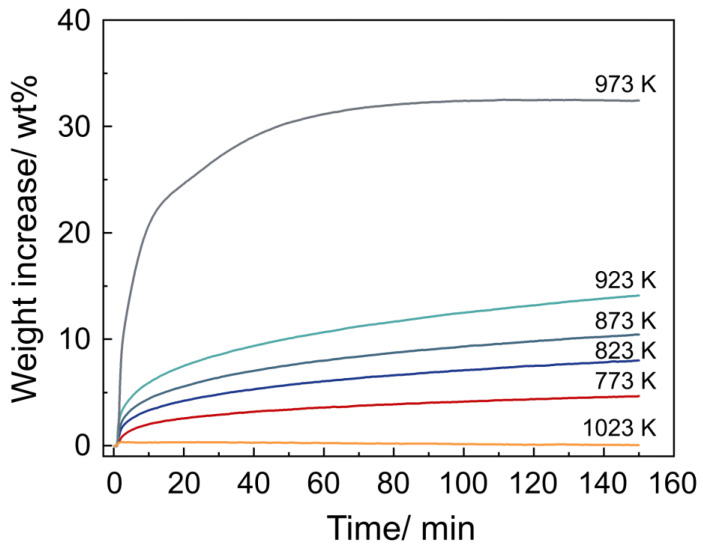
The CO_2_ adsorption curves of Li_4_SiO_4_ over time under pure CO_2_ atmosphere at different temperatures.

The influence of temperature on the CO_2_ adsorption process was analyzed in the pure CO_2_ atmosphere. Figure 2 shows the CO_2_ adsorption performance on Li_4_SiO_4_ at different temperatures. The overall weight gain of Li_4_SiO_4_ increases with the temperature in the range from 773 to 973 K. Over a duration of 150 min, the weight gain of Li_4_SiO_4_ reaches 4.95, 8.49, 11.01, and 14.84% at 773, 823, 873, and 923 K, respectively, without reaching dynamic equilibrium. However, the weight gain of Li_4_SiO_4_ reaches 32.30% at 973 K, with dynamic equilibrium being reached, which aligns with the previous research result of 33.77% [17]. We believe that these differences can be attributed to the differences in particle size. Moreover, the weight gain of Li_4_SiO_4_ drops to zero at 1023 K, indicating that the adsorption reaction does not occur at this temperature. This observation aligns with the previously reported adsorption reaction temperature range of approximately 500 to 715 °C [13]. Consequently, the optimal temperature of Li_4_SiO_4_ for CO_2_ adsorption is considered to be 973 K, with the peak adsorption capacity of 0.323 g CO_2_/g Li_4_SiO_4_. This is slightly lower than the theoretical CO_2_ adsorption capacity of 0.367 g CO_2_/g Li_4_SiO_4_, as predicted by the reaction equation Li_4_SiO_4_ + CO_2_ → Li_2_SiO_3_ + Li_2_CO_3_.

### 3.3. Phase Evolution via In Situ XRD and In Situ Raman Spectroscopy

**Figure 3:**
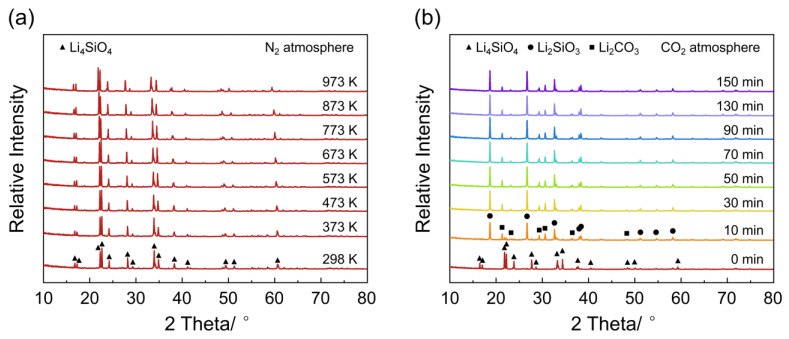
(**a**) In situ XRD patterns of Li_4_SiO_4_ heating from room temperature to 973 K in N_2_ atmosphere. (**b**) In situ XRD patterns of Li_4_SiO_4_ during CO_2_ adsorption process at 973 K in CO_2_ atmosphere.

To investigate the thermal stability of Li_4_SiO_4_, in situ XRD experiments were performed under inert N_2_ gas. Figure 3a shows the in situ XRD patterns of Li_4_SiO_4_ heating from room temperature to 973 K in N_2_ atmosphere. The XRD spectra show no significant changes during the heating process. The diffraction peaks correspond well with the standard monoclinic phase of Li_4_SiO_4_, while no diffraction peaks for crystalline Li_2_SiO_3_ or Li_2_O are observed. These indicate that the Li_4_SiO_4_ maintains the same phase when the temperature was raised from room temperature to 973 K in N_2_ atmosphere.

In order to obtain the phase transformation during the CO_2_ adsorption process of Li_4_SiO_4_, in situ XRD was performed under CO_2_ reaction conditions at 973 K. Figure 3b shows the in situ XRD patterns of Li_4_SiO_4_ during the CO_2_ adsorption process at 973 K in CO_2_ atmosphere. After the adsorption reaction at 973 K in CO_2_ atmosphere for 10 min, two groups of new peaks appear. The first group of new peaks, corresponding to the new orthorhombic phase of Li_2_SiO_3_, is observed at 18.9°, 27.0°, and 33.1°, while the second group of new peaks appears at 21.3°, 30.6°, and 31.8°, assigned to the new monoclinic phase of Li_2_CO_3_. Subsequently, as the reaction time increases, the peaks assigned to Li_4_SiO_4_ quickly disappear, and the peaks of Li_2_SiO_3_ and Li_2_CO_3_ become dominant. Moreover, no crystalline peaks of Li_2_O were observed in the in situ XRD spectra. There could be several possible reasons: (1) Li_2_O does not form during the reaction process, (2) Li_2_O, being a metastable phase, might have a very short lifespan and immediately react with CO_2_ to form Li_2_CO_3_, and (3) Li_2_O might exist in an amorphous state, which makes its detection challenging.

**Figure 4:**
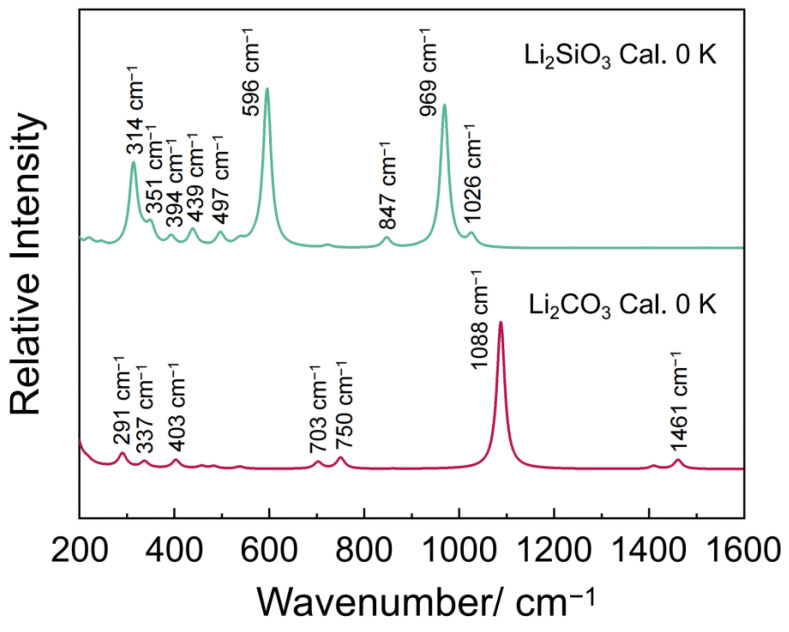
Calculated Raman spectra of Li_2_SiO_3_ and Li_2_CO_3_.

Li_2_SiO_3_ crystallizes in the orthorhombic *Ccm*2_1_ space group. The unit cell of Li_2_SiO_3_ (ICSD No. 35169) contains four formula units (Li_8_Si_4_O_12_) with lattice *a* = 5.398 Å, *b* = 9.397 Å, *c* = 4.661 Å, *α* = *β* = *γ* = 90°, and *V* = 236.209 Å^3^ [37]. As illustrated in Figure S2, the unit cell of Li_2_SiO_3_ contains four SiO_4_ tetrahedra and eight Li ions. However, unlike in Li_4_SiO_4_, the SiO_4_ tetrahedra in Li_2_SiO_3_ are not isolated; instead, they are connected to adjacent SiO_4_ tetrahedra through the bridging vertex oxygen ions, forming a chain-like structure (Figure S2). Li_2_CO_3_ crystallizes in the monoclinic *C*2/*c* space group. The unit cell of Li_2_CO_3_ (ICSD No. 16713) also contains four formula units (Li_8_C_4_O_12_) with lattice *a* = 8.359 Å, *b* = 4.977 Å, *c* = 6.194 Å, *α* = 90°, *β*= 114.72°, *γ* = 90°, and *V* = 233.778 Å^3^ [38]. As shown in Figure S3, the unit cell of Li_2_CO_3_ contains four planar covalently bonded [CO_3_]^2−^ groups and eight Li ions. According to the crystal structure of Li_2_SiO_3_ and Li_2_CO_3_, we obtained the theoretical Raman spectra of Li_2_SiO_3_ and Li_2_CO_3_ by DFT calculations. As shown in Figure 4, seven main peaks are observed at 314, 351, 394, 497, 596, 969, and 1026 in the theoretical spectrum of Li_2_SiO_3_. The bands observed at 314, 394, 497, 596, and 969 cm^−1^ are attributed to the symmetrical stretching vibrational modes of the Si–O_nb_ (where O_nb_ represents the non-bridging oxygen) bonds, while the bands located at 351 and 1026 cm^–1^ are associated with the symmetrical stretching vibrational modes of the Si–O_b_ (where O_b_ represents the bridging oxygen) bonds. Figure 4 additionally presents shows the theoretical Raman spectrum of Li_2_CO_3_. The characteristic peak of the calculated Raman spectrum of Li_2_CO_3_ is located at 1088 cm^–1^, corresponding to the symmetrical stretching vibrational modes of the C–O bonds in planar covalently bonded [CO_3_]^2−^. The major calculated vibrational modes of the Li_4_SiO_4_ and Li_2_CO_3_ are listed in Table 2.

**Figure 5:**
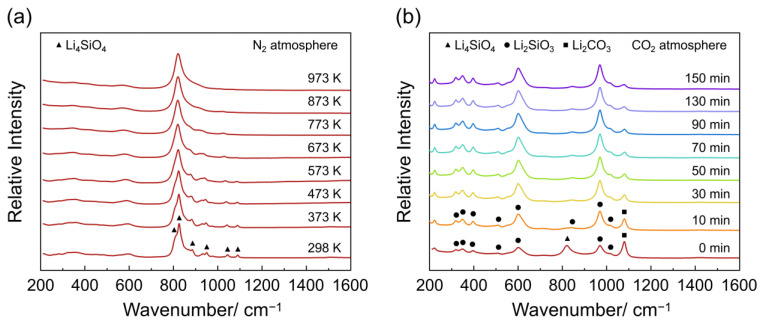
(**a**) In situ Raman spectra of Li_4_SiO_4_ heating from room temperature to 973 K in N_2_ atmosphere. (**b**) In situ Raman spectra of Li_4_SiO_4_ during CO_2_ adsorption process at 973 K in CO_2_ atmosphere.

To further confirm the in situ XRD analysis results, in situ Raman spectroscopy experiments were conducted under inert N_2_ gas and CO_2_ reaction conditions, respectively. Figure 5a shows the in situ Raman spectra of Li_4_SiO_4_ heating from room temperature to 973 K in N_2_ atmosphere. The Raman spectra of Li_4_SiO_4_ exhibit clear evidence of band broadening, with no emergence of new peaks during the heating process. This indicates that there is no phase transformation in Li_4_SiO_4_ when the temperature was heated from room temperature to 973 K in a N_2_ environment, which is consistent with the in situ XRD analysis results mentioned above. Figure 5b shows the in situ Raman spectra of Li_4_SiO_4_ during the CO_2_ adsorption process at 973 K in CO_2_ atmosphere. It can be observed that several new peaks at 221, 318, 347, 510, 601, 969, 1016, and 1080 cm^−1^ appear after the N_2_ atmosphere is switched to CO_2_ atmosphere for 10 min at 973 K. The seven peaks at 221, 318, 347, 510, 601, 969, and 1016 cm^−1^ are assigned to Li_2_SiO_3_, according to the calculated Raman spectrum of Li_2_SiO_3_. In addition, the band observed at 1080 cm^−1^ is attributed to Li_2_CO_3_. These results correspond well with the DFT calculations and previous experimental studies [39,40,41]. Subsequently, as the reaction time increases, the peaks of the Raman spectra of Li_4_SiO_4_ quickly disappear and the peaks of the Li_2_SiO_3_ and Li_2_CO_3_ phases become dominant, which is also consistent with the in situ XRD analysis results mentioned above. The results of the in situ XRD and in situ Raman spectroscopy indicate that Li_4_SiO_4_ reacted with CO_2_ to form Li_2_SiO_3_ and Li_2_CO_3_ in CO_2_ atmosphere at 973 K, with no detectable involvement of crystalline Li_2_O during the adsorption process. These findings do not support the previous assumption made by Kato et al. [15,22] that the adsorption process involves a reversible reaction between Li_2_O in the Li_4_SiO_4_ crystal structure and CO_2_.

### 3.4. Morphology of Li_4_SiO_4_ Before and After CO_2_ Adsorption

**Figure 6:**
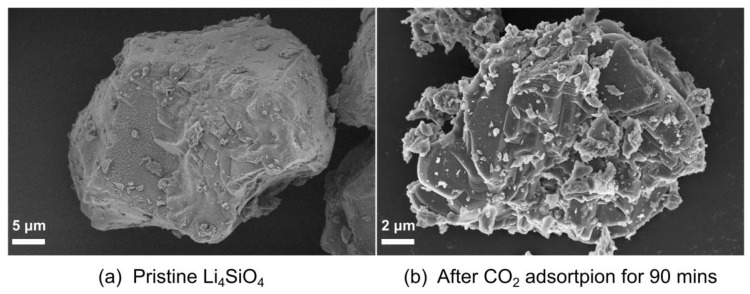
SEM images of Li_4_SiO_4_ before (**a**) and after (**b**) CO_2_ adsorption for 90 min.

Figure 6 shows the SEM images of the pristine Li_4_SiO_4_ samples before and after CO_2_ adsorption. Before CO_2_ adsorption, the sample exhibits a relatively dense and smooth surface (Figure 6a). This morphology aligns with the SEM results presented in previous studies [17], suggesting that Li_4_SiO_4_ can be considered as a non-porous material. In addition, the smooth surfaces may correspond to some specific crystallographic plane of Li_4_SiO_4_. After CO_2_ adsorption, distinct stepped structures are formed (Figure 6b), while the surface of the sample becomes looser and exhibits several minor cracks. The stepped structures were also observed by Zhang and colleagues in their research [18]. We assumed the formation of the stepped morphology may be due to the two-dimensional nucleation and growth [42] or the step-edge-guided nucleation and growth of the products [43,44].

### 3.5. CO_2_ Adsorption Mechanism of Li_4_SiO_4_

**Figure 7:**
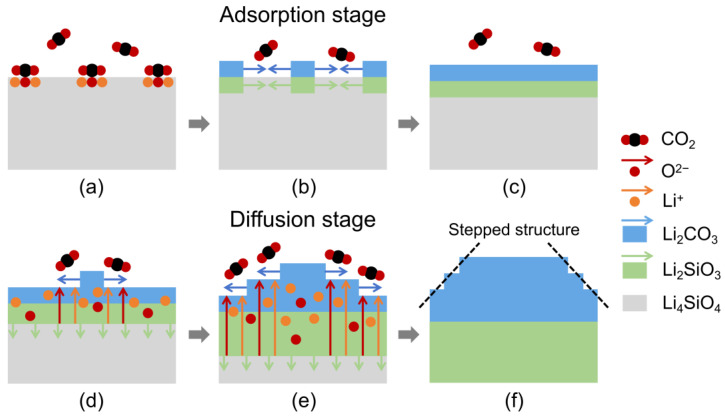
The illustration of reaction mechanism of Li_4_SiO_4_ during the CO_2_ adsorption process. (**a**) Initial CO_2_ adsorption on Li_4_SiO_4_. (**b**) Formation of Li_2_SiO_3_ and Li_2_CO_3_ nuclei. (**c**) Formation of double-shell structure. (**d**) Two-dimensional nucleation and growth of Li_2_CO_3_. (**e**) Formation of stepped structure as Li_2_CO_3_ layers continue to form. (**f**) Completion of the process as the double shells thicken.

The CO_2_ adsorption process on Li_4_SiO_4_ is generally considered to occur in two stages: initially, the adsorption stage, which is controlled by the fast surface chemical reactions, followed by the diffusion stage, governed by slower diffusion kinetics [45]. Based on the double-shell mode [17,18], we proposed a modified mode to illustrate the reaction mechanism. The whole absorption process involves the following procedures:

1. Adsorption stage: A CO_2_ molecule will be adsorbed on the Li_4_SiO_4_ surface first (Figure 7a) due to a large adsorption energy [45,46]. The C atom in CO_2_ forms a bond with a surface O^2−^ ion of Li_4_SiO_4_, and the two oxygen atoms of CO_2_ are coordinated to the two surface Li^+^ ions of Li_4_SiO_4_ [46]. Then, more adsorbed CO_2_ molecules react with Li_4_SiO_4_ to form the Li_2_SiO_3_ and Li_2_CO_3_ nuclei, as shown in Figure 7b. Subsequently, Li_2_SiO_3_ gradually grows to cover the unreacted Li_4_SiO_4_, and simultaneously, Li_2_CO_3_ forms a layer outside the Li_2_SiO_3_. This leads to the formation of a double-shell structure: the outer shell consists of Li_2_CO_3_, the inner shell is composed of Li_2_SiO_3_, and the core remains as unreacted Li_4_SiO_4_ (Figure 7c).
2. Diffusion stage: According to previous literature reports, CO_2_ molecules cannot diffuse through the Li_2_SiO_3_ and Li_2_CO_3_ shells [25]. However, Li^+^ and O^2−^ ions, which are formed by the unreacted Li_4_SiO_4_ at the interface between Li_2_SiO_3_ and the remaining unreacted Li_4_SiO_4_, are capable of diffusing through these two shells [25,47,48]. As the reaction proceeds, the Li^+^ and O^2−^ ions react with CO_2_ molecules at the surface of the existing Li_2_CO_3_ layer, leading to the two-dimensional nucleation and growth of Li_2_CO_3_, as shown in Figure 7d. In a similar manner, new two-dimensional nuclei of Li_2_CO_3_ continue to form on the surface of the newly generated Li_2_CO_3_. This process results in different layers of Li_2_CO_3_, which form a stepped structure, and growth of Li_2_CO_3_ persists along the edges of these steps, as depicted in Figure 7e. Finally, the double shells become thicker until the reaction is fully completed (Figure 7f).

## 4. Conclusions

In conclusion, the Li_4_SiO_4_ absorbent was successfully synthesized by solid-state reaction. The maximum CO_2_ adsorption capacity of Li_4_SiO_4_ in CO_2_ atmosphere at 973 K reaches 0.323 g CO_2_/g Li_4_SiO_4_, which is slightly lower than the theoretical value of 0.367 g CO_2_/g Li_4_SiO_4_. To elucidate the CO_2_ adsorption mechanism of Li_4_SiO_4_, in situ XRD and in situ Raman spectroscopy were used to study the phase transformations during the CO_2_ adsorption process. We observed that Li_4_SiO_4_ reacted with CO_2_ to form Li_2_SiO_3_ and Li_2_CO_3_, with no detectable involvement of crystalline Li_2_O during the adsorption process. SEM images further characterized the morphological changes in the Li_4_SiO_4_ surface before and after CO_2_ adsorption, with the formation of stepped structures. Finally, a modified double-shell mechanism was proposed to elucidate the underlying mechanism of the CO_2_ adsorption process. The possible cause of the formation of stepped structures might be attributed to the two-dimensional nucleation and growth of Li_2_CO_3_; this hypothesis, however, requires further investigation.

## Figures and Tables

**Table 1:** The assignment of major vibration modes of crystalline Li_4_SiO_4_.

Wavenumber		Vibration Modes	Type of Vibration
*ʋ*_exp_ (cm^−1^)	*ʋ*_cal_ (cm^−1^)		
800	779	*A_g_*	*ʋ_s_*(Si–O)
817	811	*A_g_*	*ʋ_s_*(Si–O)
862	865	*B_g_*	*ʋ_r_*(Si–O)
876	883	*A_g_*	*ʋ_s_*(Si–O)
924	920	*A_g_*	*ʋ_s_*(Si–O)
941	933	*A_g_*	*ʋ_s_*(Si–O)
967	951	*B_g_*	*ʋ_r_*(Si–O)
1036	1003	*A_g_*	*ʋ_s_*(Si–O)
1082	1045	*A_g_*	*ʋ_s_*(Si–O)

*ʋ*_s_ and *ʋ*_r_ represent the symmetric stretching vibrations and the rocking vibrations.

**Table 2:** The assignment of major vibration modes of crystalline Li_4_SiO_4_ in carbon dioxide atmosphere at 973 K.

Wavenumber		Vibration Modes	Type of Vibration
*ʋ*_exp_ (cm^−1^)	*ʋ*_cal_ (cm^−1^)		
318	351	*A*_2_	*ʋ_s_* (Si–O_b_)
347	394	*A*_1_	*ʋ_s_* (Si–O_nb_)
396	439	*A*_1_	*ʋ_s_* (Si–O_nb_)
510	497	*A*_1_	*ʋ_s_* (Si–O_nb_)
601	596	*A*_1_	*ʋ_s_* (Si–O_nb_)
705	703	*A*_g_	*ʋ_s_* (C–O)
729	750	*B*_g_	*ʋ_r_* (C–O)
842	847	*A*_1_	*ʋ_s_* (Si–O_nb_)
969	969	*A*_1_	*ʋ_s_* (Si–O_nb_)
1016	1026	*A*_2_	*ʋ_s_* (Si–O_b_)
1080	1088	*A*_g_	*ʋ_s_* (C–O)
1410	1461	*A*_g_	*ʋ_s_* (C–O)

*ʋ*_s_ and *ʋ*_r_ represent the symmetric stretching vibrations and the rocking vibrations.

## Data Availability

The original contributions presented in this study are included in the article/Supplementary Material. Further inquiries can be directed to the corresponding author.

## Supplementary Materials

The following supporting information can be downloaded at: https://www.mdpi.com/article/10.3390/ma18020319/s1, Figure S1: Schematic representation of the Li_4_SiO_4_ crystal structure. Key: SiO_4_, blue tetrahedra; lithium, green. Partially occupied Li sites are represented by partial shading. Unit cells are shown as dotted lines. Figure S2: Schematic representation of the Li_2_SiO_3_ crystal structure. Key: SiO_4_, blue tetrahedra; lithium, green. Unit cells are shown as dotted lines. Figure S3: Schematic representation of the Li_2_CO_3_ crystal structure. Key: CO_3_, brown triangle; lithium, green. Unit cell is shown as dotted lines.
