# Supplementary material for: Mechanistic Insights into CO2 Adsorption of Li4SiO4 at High Temperature
Source: Materials (Basel). 2025 Jan 12;18(2):319. doi: 10.3390/ma18020319 (PMC11766996; doi:10.3390/ma18020319)
Supplement: Supplementary file 1 [file materials-18-00319-s001.zip › materials-3412454-supplementary.pdf]

Supplementary Materials: Mechanistic Insights into CO_2 Adsorption of Li_4SiO_4 at High Temperature

Nan Ma [1,2,*], Silin Wei [1], Jinglin You [2], Fu Zhang [3] and Zhaohui Wu [1]

[1] Hunan Key Laboratory of Applied Environmental Photocatalysis, School of Materials and Environmental Engineering, Changsha University, Changsha 410022, China; w1625272361@163.com (S.W.); hubeiwzh1624@126.com (Z.W.)
[2] State Key Laboratory of Advanced Special Steel, Shanghai Key Laboratory of Advanced Ferrometallurgy, School of Materials Science and Engineering, Shanghai University, Shanghai 200444, China; jlyou@163.com
[3] State Key Laboratory of Applied Organic Chemistry, Key Laboratory of Nonferrous Metal Chemistry and Resources Utilization of Gansu Province, College of Chemistry and Chemical Engineering, Lanzhou University, Lanzhou 730000, China; fuzhang201026@163.com
* Correspondence: doria_mn@163.com

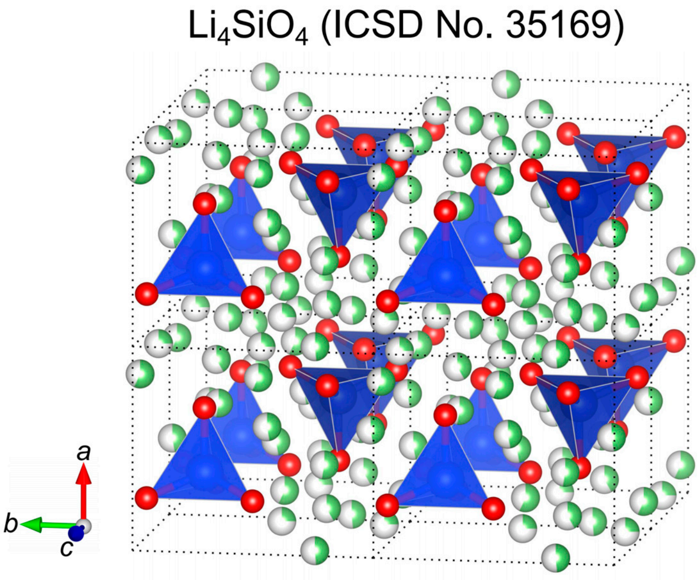

Figure S1. Schematic representation of the Li_4SiO_4 crystal structure. Key: SiO_4, blue tetrahedra; lithium, green. Partially occupied Li sites are represented by partial shading. Unit cells are shown as dotted lines.

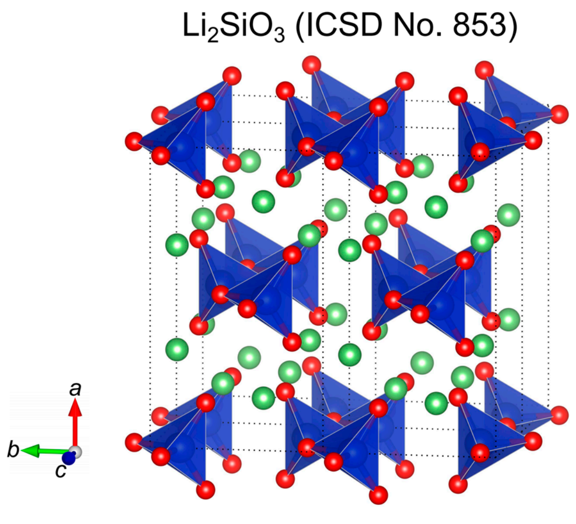

Figure S2. Schematic representation of the Li_2SiO_3 crystal structure. Key: SiO_4, blue tetrahedra; lithium, green. Unit cells are shown as dotted lines.

Li_2CO_3 (ICSD No. 16713)

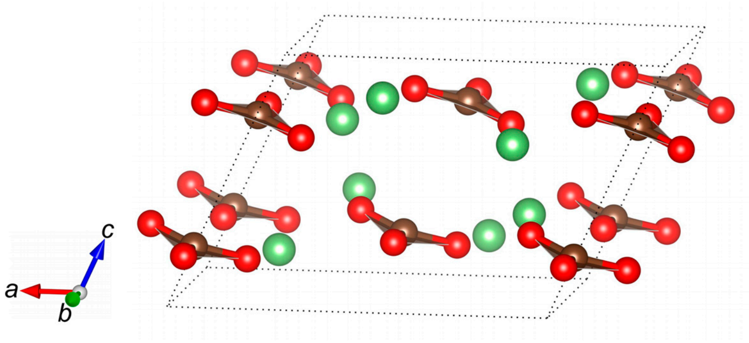

Figure S3. Schematic representation of the Li_2CO_3 crystal structure. Key: CO_3, brown triangle; lithium, green. Unit cell is shown as dotted lines.
